# The mediating role of menstrual irregularity on obesity and sexual function in Chinese women with pelvic floor disorders: a cross-sectional study

**DOI:** 10.1186/s12905-023-02594-8

**Published:** 2023-08-31

**Authors:** Xiaoyang Lv, Huijun Yang, Miaomiao Yan, Xuli Jin, Xin Shen, Shu Li, Miqing Zhang, Sha Su, Xiaoyan Liu, Jie Chen

**Affiliations:** 1 Department of Maternal and Child Health, School of Public Health, Cheeloo College of Medicine, Shandong University, Jinan, Shandong 250012 China; 2 China Center for Health Development Studies, Peking University, Beijing, China; 3 Key Laboratory of Birth Regulation and Control Technology of National Health Commission of China, Shandong Provincial Maternal and Child Health Care Hospital, Jinan, Shandong 250014 China; 4 Yichang Hospital of Traditional Chinese Medicine, Yichang, Hubei 443000 China; 5 Women’s Pelvic Floor Functional Health Center, Yunshi Health Industry, Jinan, Shandong 250000 China; 6 Department of Obstetrics and Gynecology, Qilu Hospital, Shandong University, Jinan, Shandong 250012 China

**Keywords:** Female sexual function, Obesity, Menstrual irregularity, Mediating Effect

## Abstract

**Background:**

Sexual problems are common among women with pelvic floor disorders (PFD). Few studies have explored the relationship between obesity and sexual function in women with PFD. This study aimed to prove that obesity was a risk factor for worse sexual function in women with PFD, and to investigate the mediating role of menstrual irregularity.

**Methods:**

This was a cross-sectional study involving 783 women with PFD from Shandong Province, China between June 2020 and February 2021. Female sexual function was assessed using the Pelvic Organ Prolapse/UI Sexual Questionnaire-12 (PISQ-12). Obesity was defined as BMI ≥ 28.0. Menstrual irregularity was defined as menstrual cycles ≥ 35 or menstrual cycles < 25 days. Logistic regression and multiple linear regression were employed to explore the association among obesity, menstrual irregularity and sexual function.

**Results:**

Obesity was associated with worse PISQ-12 scores compared with normal- weight women (mean score 28.14 ± 7.03 versus 32.75 ± 5.66, p < 0.001). After adjusting for controlling variables, women with obesity (β= -3.74, p < 0.001) and menstrual irregularity (β= -3.41, p < 0.001) had a worse sexual function. Menstrual irregularity had a mediation effect on the association between obesity and sexual function.

**Conclusions:**

This study provided evidence that obesity was associated with worse sexual function in women with PFDs, and the effect of obesity on sexual function was partially mediated by menstrual irregularity. Weight control may have potential benefits for improving sexual function and preventing female sexual dysfunction. It’s also important to pay attention to the menstrual cycle.

## Introduction

Sexual health is a universal part of women’s life quality at all ages. If a woman has sexual dysfunction, it can be dyspareunia, lack of vaginal lubrication, difficulty to reach orgasm, vaginal bleeding or irritation after sex, and loss of sexual desire [1, 2], and their quality of life will be seriously affected. The prevalence of sexual dysfunction in women is between 38% and 43% [3, 4]. Studies have found that sexual problems are common among women with pelvic floor disorders (PFD) [5]. Many studies found a significantly higher prevalence of sexual dysfunction in patients complaining of PFD women [6, 7].Women with more severe PFD were significantly more likely to report decreased arousal (P < 0.01), infrequent orgasm (P < 0.01), and increased dyspareunia (P < 0.01) [5]. Although several approaches are able for the management of POP and improve PISQ-12 scores, the best strategy in case of recurrence after vaginal vault prolapse still remains debated [8, 9]. There is a high prevalence of sexual dysfunction in women with urinary incontinence, one of the PFDs, and more than 40% of them had sex-life impairment [10]. There is much literature on sexual function and sexual dysfunction in women with PFD, but few evidence on the influencing factors of sexual function in them.

Research results showed that body mass index (BMI) and amenorrhea were associated with sexual function [11, 12]. Some studies show a possible causal relationship between obesity and erectile dysfunction in men, but theexact nature of the association between obesity and female sexual dysfunction has yet to be determined [13]. Obese people have a high BMI. There is a growing body of literature regarding the impact of BMI on sexual function in women, with conflicting findings. Coelho et al. found that a high BMI was associated with risk factors for female sexual dysfunction [14]. Kolotkin et al. also suggested that higher BMI was associated with greater impairments in sexual quality of life and obesity is associated with a lack of enjoyment of the sexual activity, lack of sexual desire, difficulties with sexual performance, and avoidance of sexual encounters [13]. However, some studies have shown sexual functioning is not related to obesity in women [15, 16].

Studies have shown positive associations between BMI and menstrual irregularity in women [17, 18]. Furthermore, a large cohort study demonstrated that overweight and obese women had higher odds of menstrual irregularity than women who were underweight/normal weight [19]. Obesity can be accompanied by several neuroendocrine and ovarian dysfunctions, including menstrual irregularity [20]. Other studies showed sexual desire and activity are also higher during ovulation and those women who have menstrual irregularity are more likely to suffer from sexual dysfunction [12, 21].

Few studies have explored the relationship between obesity and sexual function in women with PFD. Obese adolescents with menstrual irregularities often have polycystic ovary syndrome, and these patients have underlying abnormal sexual behavior [22]. In addition, previous studies focused more on the effects of obesity rather than irregular menstruation on sexual function and no discussed the mediating effect between them except us. The purpose of this study was to prove that obesity is a factor influencing sexual function and to explore the mediating effect of menstrual irregularities on the relationship between obesity and sexual function in women with PFD. This study attempted to prevent the decline in the quality of life of PFD patients and provide effective information for the prevention of subsequent sexual dysfunction in PFD patients.

## Materials and methods

### Participants

We conducted a cross-sectional study including 842 women seeking care for PFDs as new consultations at the Postpartum Health Recovery Center in Shandong, China. between June 2020 to February 2021. PFD women with urinary incontinence (UI), pelvic organ prolapse (POP) and/or fecal incontinence were included. UI was assessed using the International Consultation on Incontinence Questionnaire-Urinary Incontinence Short Form (ICIQ-UI SF), which has been prepared and authorized for use by the International Advisory Committee on UI. POP and fecal incontinence were the participants who claimedto be diagnosed by the clinician.

The inclusion criteria were as follows: [1] women aged 20–45 who have given birth; [2] have PFD; [3] able to understand the contents of the questionnaire and complete the questionnaire autonomously. The exclusion criteria were as follows: [1] sexual dysfunction caused by genital malformation; [2] complete loss of nerve control function in the pelvic floor. Participants were surveyed about their medication and surgical history and they were not on hormone therapy for menstrual disorders.

Questionnaire investigators were staff who received formal training. The investigator explained the relevant situation to the respondent before the survey and filled it out after obtaining their consent and explaining that their privacy would be fully protected. After data screening, data from 783 respondents were included in the analysis. The effective rate of the questionnaire was 93.0%.

### Variables

#### Dependent variable

##### Sexual function

Sexual function is assessed using the Chinese version of the Pelvic Organ Prolapse/UI Sexual Questionnaire-12 (PISQ-12). It has been verified that the PISQ-12 has an adequate and high internal consistency (Cronbach α = 0.725) and a high test–retest reliability (intraclass correlation coefficient = 0.745; P<0.01) in Chinese women with PFD [23, 24] and has been regarded as a high reliability and validity in the Chinese population and highly recommended for clinical treatment and research. The PISQ-12 consists of 12 items, including three aspects: affective factors, physiological factors, and sexual partner factors. Each item is scored from 0 to 4 points, which represent always, often, sometimes, rarely, and never. There are four reverse scoring questions: “always” is 4 points, “often” is 3 points, “sometimes” is 2 points, “rarely” is 1 point, and “never” is 0. The scale has a total score of 48 points. The lower the PISQ-12 score, the lower the quality of sexual life and the worse the sexual function.

#### Independent variable

##### Socio-demographic characteristics

Socio-demographic characteristics included five items: age, household registration, employment, household income, and education. Age was divided into five groups, respectively, ≤ 25 years, 26–30 years, 31–35 years, 35–40 years, and > 40 years. Household registration included urban and rural. Employment and medical insurance status were classified as yes and no. The annualincome included less than 20,000¥, 20,000 to 50,000¥, 50,000 to 100,000¥, and more than 100,000¥. Education included junior high school and below, high school, college and above.

##### Obesity

Women reported their height and weight and BMI was calculated as weight (kg)/height(m)^2^ and categorized as: underweight (BMI < 18.5), normal (BMI 18.5–23.9), Overweight (BMI 24.0-27.9) and obese (BMI ≥ 28.0). For the analyses, obesewomen were defined as having BMI ≥ 28.0.

##### Menstrual irregularity

Menstrual cycle length was the time from the first day of one period to the first day of the next and participants were questioned on the length of their usual menstrual cycle. Menstrual irregularity was defined as yes (menstrual cycles ≥ 35 or < 25 days) or no (25 days ≤ menstrual cycles < 35 days). Participants were surveyed about their medication and surgical history and they were not on hormone therapy for menstrual disorders.

##### Obstetric factors

Obstetric factors included: number of deliveries, delivery mode, pregnancy complications, history of pelvic surgery. In our surveys, women were asked: ‘Have you had children before?’ Women who reported yes were categorized as “number of deliveries > 1” and reported no were categorized as “number of deliveries = 1”. Delivery mode was divided into vaginal and cesarean deliveries. Pregnancy complications and history of pelvic surgery as categories were coded as yes/no.

### Statistical analyses

First, descriptive statistics (frequencies and percentages) were used to summarise the participants’ characteristics. The PISQ-12 scores across different subgroups of categorical variables were presented by the mean and standard deviation, and were compared using t-tests or analysis of variance.

Then, we performed the following analysis to explore the association among obesity, menstrual irregularity and sexual function in women with PFD and to further explore the mediation effect of menstrual irregularity on the association between obesity and sexual function:

1. Using logistic regression to estimate the association between obesity and menstrual irregularity.
2. Using multiple linear regression to estimate the association between obesity and sexual function.
3. Using multiple linear regression to estimate the association between obesity and sexual function when menstrual irregularity was included.

All analyses were conducted using SPSS Statistics version 25. Statistical significance was set at P ≤ 0.05.

### Ethical considerations

The Ethical Committee of Shandong University reviewed and approved the study protocol (IRB number: LL20210704). All participants completed an informed consent form.

## Results

### Participants characteristics

The 783 women with PFD included in the study were mainly aged 25–40 years (81.5%). Most women had rural household registration (63.9%), were employed (78.3%), and did not have a university degree (65.8%). Their annual household income was mainly 20,000–50,000¥ (32.7%) and 50,000-100,000¥ (32.7%).

The majority had normal BMI (65.9%), 11.6% were underweight, 15.2% were overweight and 7.3% were obese. Most also reported having regular menstruation (72.9%), giving birth for the first time (59.6%) and vaginal delivery (68.9%). Few women had pregnancy complications or a history of pelvic surgery.

The average score of the PISQ-12 was 32.09, with a standard deviation of 5.82. The results showed that there were statistically significant differences in the PISQ-12 score of survey subjects with different household registration, household income, education level, BMI, and menstrual irregularity. Women with rural household registration, low annual household income, low education level, obesity and menstrual irregularity scored lower and had poorer sexual function (P < 0.05). Obesity was associated with worse PISQ-12 scores compared with normal weight women (mean score 28.14 ± 7.03 versus 32.75 ± 5.66). (Table 1).

**Table 1:** PISQ-12 score of Women with different sample characteristics

Variable	N (%)	PISQ-12 score	t/F	P
Age				
< 25	53(6.8)	32.58 ± 6.31	0.796	0.528
25–30	278(35.5)	31.95 ± 6.04		
31–35	249(31.8)	31.99 ± 5.56		
36–40	111(14.2)	32.84 ± 5.46		
>40	92(11.7)	31.57 ± 6.00		
**Household registration**				
rural	500(63.9)	31.68 ± 5.91	-2.589	0.010*
urban	283(36.1)	32.80 ± 5.60		
**Employment**				
No	170(21.7)	31.77 ± 5.91	-0.804	0.422
Yes	613(78.3)	32.18 ± 5.80		
**Household income**				
≤ 20,000	149(19)	30.83 ± 5.87	4.187	0.006*
20,000–50,000	256(32.7)	32.19 ± 5.66		
50,000-100,000	256(32.7)	32.13 ± 5.90		
≥ 100,000	122(15.6)	33.31 ± 5.70		
**Education**				
Junior high school and below	230(29.4)	31.27 ± 5.71	6.043	0.002*
High school	285(36.4)	31.86 ± 5.92		
University and above	268(34.2)	33.03 ± 5.70		
**Number of deliveries**				
1	467(59.6)	32.14 ± 5.71	0.174	0.862
> 1	316(40.4)	32.06 ± 5.90		
**Delivery mode**				
Vaginal delivery	539(68.9)	31.68 ± 5.85	-1.331	0.184
Cesarean section	244(31.1)	32.27 ± 5.80		
**Pregnancy complications**				
No	737(94.1)	32.15 ± 5.84	1.233	0.218
Yes	46(5.9)	31.06 ± 5.45		
**History of pelvic surgery**				
No	644(82.2)	32.03 ± 5.76	-0.581	0.561
Yes	139(17.8)	32.35 ± 6.09		
**BMI**				
normal	516(65.9)	32.75 ± 5.66	9.973	0.000*
underweight	91(11.6)	31.92 ± 5.70		
overweight	119(15.2)	30.53 ± 5.70		
obese	57(7.3)	28.14 ± 7.03		
**Menstrual irregularity**				
No	571(72.9)	33.09 ± 5.41	9.398	0.002
Yes	212(27.1)	29.42 ± 6.07		

Note: ***BMI*** body mass index, *p < 0. 05

### The mediation effect of menstrual irregularities on the association between obesity and sexual function

#### The association between obesity and menstrual irregularity

Logistic regression showed that obese (OR = 2.54, p = 0.028) was significantly associated with menstrual irregularity after adjusting for control variables such as age, household registration, employment, household income, education, number of deliveries, delivery mode, pregnancy complications and history of pelvic surgery. Obesity increased the risk of menstrual irregularity. (Table 2)

**Table 2:** Logistic regression model of factors related to menstrual irregularity

Group	B	Standard error	Wald	OR(95%CI)	*P*
Age					
< 25				1	
25–30	0.651	0.38	2.94	1.12(0.91,4.03)	0.086
31–35	-0.147	0.281	0.274	0.86(0.49,1.49)	0.601
36–40	-0.094	0.281	0.112	0.91(0.53,1.58)	0.738
>40	-0.580	0.34	2.901	0.56(0.29,1.09)	0.089
**Household registration**					
rural				1	
urban	-0.110	0.187	0.346	0.89(0.62,1.29)	0.556
**Employment**					
No				1	
Yes	-0.028	0.208	0.019	0.97(0.64,1.46)	0.892
**Household income**					
≤ 20,000				1	
20,000–50,000¥	-0.022	0.234	0.009	0.97(0.61,1.54)	0.925
50,000-100,000¥	-0.244	0.243	1.006	0.78(0.48,1.26)	0.316
≥ 100,000	-0.448	0.303	2.19	0.63(0.35,1.15)	0.139
**Education**					
Junior high school and below				1	
High school	-0.069	0.211	0.106	0.93(0.61,1.41)	0.744
university and above	-0.443	0.235	3.554	0.64(0.40,1.01)	0.059
**Number of deliveries**					
1				1	
> 1	-0.184	0.182	1.016	0.83(0.58,1.18)	0.313
**Delivery mode**					
Vaginal delivery					
Cesarean section	0.192	0.181	1.127	1.21(0.85,1.72)	0.288
**Pregnancy complications**					
No				1	
Yes	-0.086	0.355	0.058	0.91(0.45,1.84)	0.809
**History of pelvic surgery**					
No				1	
Yes	-0.644	0.243	7.057	0.52(0.32,0.84)	0.008*
**BMI**					
normal				1	
underweight	-0.494	0.278	3.167	0.61(0.35,1.05)	0.075
overweight	0.086	0.221	0.151	1.08(0.70,1.67)	0.698
obese	0.932	0.423	4.854	2.53(1.10,5.81)	0.028*

Note: ***BMI*** body mass index, ***B*** unstandardized regression coefficient, ***CI*** confidence interval,*p < 0. 05

#### The association between obesity and sexual function

Multiple linear regression showed that obesity (β= -4.44, p < 0.001) was significantly associated with sexual function after adjusting for control variables. Obese Women had a worse PISQ-12 score compared with normal-weight women, which means worse sexual function. (Table 3)

**Table 3:** The mediating effect of menstrual irregularity on the association between obesity and sexual function

Characteristics	Model without menstrual irregularity			Model with menstrual irregularity		
	B	se	P	B	se	P
Age						
< 25						
25–30	-1.13	0.86	0.19	-1.74	0.84	0.04
31–35	-0.95	0.89	0.28	-1.53	0.86	0.08
36–40	-0.12	0.99	0.91	-0.98	0.96	0.31
>40	-1.30	1.02	0.20	-1.82	0.98	0.07
**Household registration**						
rural						
urban	0.65	0.45	0.15	0.58	0.44	0.18
**Employment**						
No						
Yes	-0.34	0.52	0.51	-0.36	0.50	0.47
**Household income**						
≤ 20,000						
20,000–50,000¥	1.22	0.60	0.04	1.20	0.58	0.04
50,000-100,000¥	0.94	0.61	0.12	0.78	0.59	0.18
≥ 100,000	1.91	0.72	0.01	1.63	0.70	0.02
**Education**						
Junior high school and below						
High school	0.32	0.53	0.55	0.27	0.51	0.59
university and above	1.45	0.57	0.01	1.17	0.55	0.03
**Number of deliveries**						
1						
> 1	0.21	0.45	0.64	0.10	0.43	0.82
**Delivery mode**						
Vaginal delivery						
Cesarean section	0.55	0.45	0.22	0.43	0.43	0.32
**Pregnancy complications**						
No						
Yes	-0.48	0.88	0.59	-0.54	0.85	0.53
**History of pelvic surgery**						
No						
Yes	0.50	0.53	0.35	0.12	0.52	0.82
**BMI**						
normal						
underweight	-0.62	0.63	0.32	-0.92	0.61	0.13
overweight	-2.09	0.55	0.00	-2.03	0.53	0.00
obese	-4.44	1.14	0.00*	-3.74	1.11	0.00*
**menstrual irregularity**						
	-	-	-	-3.41	0.45	0.00*

Note: ***BMI*** body mass index, ***B*** unstandardized regression coefficient, ***se*** standard error, *p < 0. 05

#### Analysis of mediation effect model

**Fig. 1:**
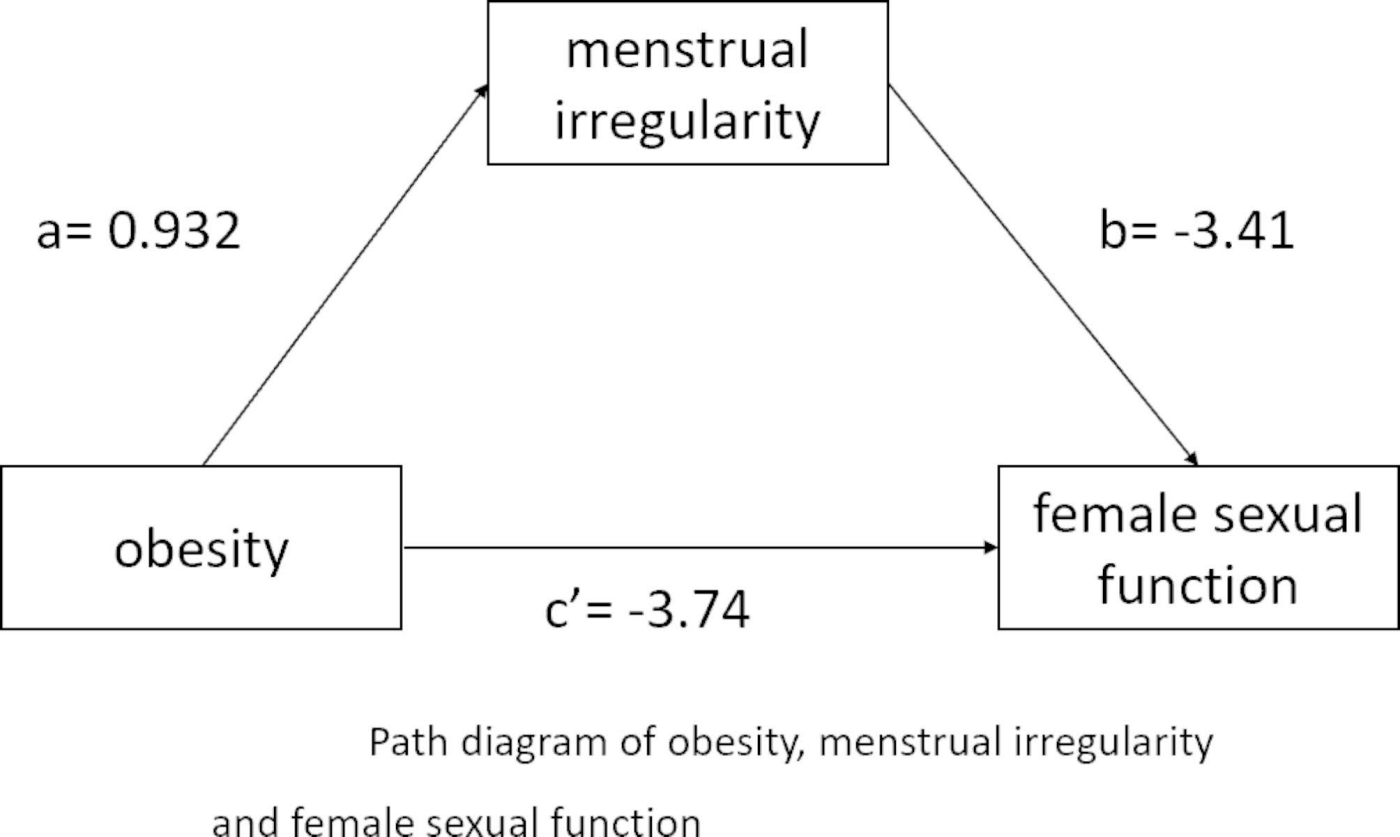
Path diagram of obesity, menstrual irregularity and female sexual function. **Note**: Female sexual function was assessed by PISQ-12 score. The ‘ **a** ’ shows the effect of obesity on menstrual irregularity and the ‘ **b** ’ shows the effect of menstrual irregularity on the female sexual function. The ‘ **c**’ ’ shows the direct effect of BMI on female sexual function

After menstrual irregularity was included in the model, multiple linear regression showed that obese (β= -3.74, p < 0.001) was still significantly associated with sexual function after adjusting for control variables. The regression coefficient of obesitychanged from − 4.44 to -3.74. At the same time, menstrual irregularity (β= -3.41, p < 0.001) was significantly related to sexual function, and women with menstrual irregularity are more likely to have worse sexual function. Therefore, menstrual irregularity had a mediation effect on the association between obesity and sexual function. (Table 3; Fig. 1)

## Discussion

The present study aimed at investigating the relationship between obesity and sexual function in women with PFD. To date, the relationship has been understudied and may have important implications for understanding. In this study, we proved that obesity is an influencing factor in sexual function among women with PFD in China. Women with obesity were more likely to have worse sexual function compared with normal-weight women. In order to further explore the mechanism, we innovatively added menstrual irregularity to the model to explore the possibility that the relationship between obesity and sexual function in women may be mediated by menstrual irregularity.

The association between obesity and sexual dysfunction in women has been supported by some findings but only partially in others. In our hypothesis, we expected that obese women would have worse sexual function and a worse sexual function compared to normal-weight women. This hypothesis was confirmed. This finding is consistent with literature showing that higher BMI was associated with greater impairments in sexual quality of life [13]. Tsai’s questionnaire survey also showed that obesity is associated with worse sexual function in women with PFD [25].

Research data suggest that the relationship between sexual function and obesity in women is highly complex and influenced by multiple factors. Obesity may cause sexual dysfunction by affecting psychological and physiological pathways. Obesity is a major risk factor for many chronic medical conditions, including diabetes mellitus, hypertension, and depression. Many of these conditions and their associated treatments may be associated with sexual dysfunction [26]. For example, type 2 diabetes, a common complication of overweight patients, renders secretion and metabolism disorders more serious, causing sexual dysfunction [27]. For women with pelvic floor dysfunction, obesity may cause the deterioration of sexual function due to the weakening of the superficial transverse muscles that fix the central tendon of the perineum, the entire perineal body droops, the pudendal nerve is damaged, and the pelvic floor muscles relax, which affects normal sexual function [28]. Several investigations pointed out the necessity of considering psychological factors when the relationship between BMI and female sexual functioning is evaluated [12]. Di Nardo et al. supported the notion that sexual functioning is not related directly to BMI in women but to more complex interactions of body weight, satisfaction with one’s own body image, and levels of self-esteem [29].

Some scholars think the effect that menstrual irregularity brings may be concerned with hormone levels. In addition, both hypothyroidism and hyperthyroidism are associated with changes in concentrations of sex hormones in both sexes, ovulatory function (menstrual irregularities, menarche and menopause) in women [30]. Some investigation show that ovarian steroids appear to modulate the sexual activity of women [31]. Studies conducted with surgically menopausal women reported a marked decline in sexual desire which may be reversed by hormonal therapy providing evidence that hormones affect female sexuality [32, 33]. Previous studies have shown that older age is a risk factor for female sexual dysfunction [34, 35]. A study included 15,048 heterosexual women aged 16–74 years have revealed that all sexual dysfunctions are significantly associated with age in women. Most sexual dysfunctions increase with age, and some display a U-shaped association with age [36]. Juliato et al. believe that the increased prevalence of female sexual dysfunction among middle-aged and elderly women may be related to ageing or menopause and estrogen level decreased. Therefore, some studies believe that menopause is an independent factor that causes female sexual dysfunction, and age is only a confounding factor [37, 38]. We found that women with menstrual irregularity were a factor affecting sexual function, which may also support the above view.

This study proved that obesity was associated with worse sexual function in women with PFDs, and explored the mediating role of menstrual irregularity in the association between obesity and sexual function. It provides a new idea for exploring the influence of obesity on female sexual function in the future. Due to the high and rising prevalence of obesity, we are concerned that difficulties with sexual functioning are also becoming more common in women with PFD. The silver lining is that many studies show that sexual functioning can be improved with weight loss [13]. Our results may have implications for women with PFD that weight control may have potential benefits for improving sexual function and preventing female sexual dysfunction. It’s also important to pay attention to the menstrual cycle. Accumulating lines of evidence already highlighted that PFD has a severe impact on quality of life and may lead to a significant impairment of sexual functions [39]; for this reason, a multidisciplinary approach is recommended for an accurate management in the future [40].

There were some limitations in this study. First, the data we used was cross-sectional, thus the causal relationship between variables could not be determined. And some key influencing factors, such as the time of delivery and the presence or absence of ovarian hypofunction, are factors that affect sexual dysfunction, but we did not take this into account in the study design. Second, the subjects were women with PFD, and the result could not be easily extended to the general population. Third, the partner’s experiences were not assessed, as sexual life depends on two persons, and getting information from only the female partner is unilateral. Finally, we verified only one mediation variable in this study, more potential mechanisms need to be explored in the future.

## Conclusion

This study provided evidence that obesity was associated with worse sexual function in women with PFDs, and the effect of obesity on sexual function was partially mediated by menstrual irregularity. Weight control may have potential benefits for improving sexual function and preventing female sexual dysfunction. It’s also important to pay attention to the menstrual cycle.

## Data Availability

The datasets generated and analysed during the current study are not publicly available due to privacy restrictions but are available from the corresponding author on reasonable request.
